# The genome sequence of the comb-clawed beetle, *Prionychus ater* (Fabricius, 1775) (Coleoptera: Tenebrionidae)

**DOI:** 10.12688/wellcomeopenres.24801.1

**Published:** 2025-09-08

**Authors:** Dmitry Telnov, Svetlana Nikolaeva, Maxwell V. L. Barclay, Natural History Museum Genome Acquisition Lab, Darwin Tree of Life Barcoding Collective, Wellcome Sanger Institute Tree of Life Management, Samples and Laboratory team, Wellcome Sanger Institute Scientific Operations: Sequencing Operations, Wellcome Sanger Institute Tree of Life Core Informatics team, Tree of Life Core Informatics collective, Darwin Tree of Life Consortium

**Affiliations:** 1 Natural History Museum, London, England, UK; 2 Daugavpils University, Daugavpils, Latvia; 3 University of Latvia, Institute of Biology, Riga, Latvia; 4 Russian Academy of Sciences, Borissiak Paleontological Institute, Moscow, Russian Federation

**Keywords:** Prionychus ater; comb-clawed beetle; genome sequence; chromosomal; Coleoptera

## Abstract

We present a genome assembly from an individual male *Prionychus ater* (comb-clawed beetle; Arthropoda; Insecta; Coleoptera; Tenebrionidae). The assembly contains two haplotypes with total lengths of 385.22 megabases and 347.54 megabases. Most of haplotype 1 (97.6%) is scaffolded into 14 chromosomal pseudomolecules, including the X sex chromosome. Haplotype 2 was assembled to scaffold level. The mitochondrial genome has also been assembled, with a length of 16.26 kilobases. This assembly was generated as part of the Darwin Tree of Life project, which produces reference genomes for eukaryotic species found in Britain and Ireland.

## Species taxonomy

Eukaryota; Opisthokonta; Metazoa; Eumetazoa; Bilateria; Protostomia; Ecdysozoa; Panarthropoda; Arthropoda; Mandibulata; Pancrustacea; Hexapoda; Insecta; Dicondylia; Pterygota; Neoptera; Endopterygota; Coleoptera; Polyphaga; Cucujiformia; Tenebrionoidea; Tenebrionidae; Alleculinae; *Prionychus*; *Prionychus ater* (Fabricius, 1775) (NCBI:txid347445)

## Background

The Tenebrionidae Latreille, 1802, commonly known as darkling beetles, is the seventh largest family of the order Coleoptera with more than 30 000 species in over 2 300 valid genera in 11 subfamilies worldwide ( Bouchard *et al.*, 2021) and is described as “hyperdiverse” ( Kergoat *et al.*, 2014). Adult tenebrionids are extremely heterogenous morphologically: nearly every unusual looking specimens one is not able to arrange to a family is highly likely to be a darkling beetle. On the contrary, immature tenebrionids (often called “mealworms”) are quite uniform in their appearance. The family is of cosmopolitan distribution, most speciose in the tropical and arid subtropical regions. Darkling beetles are found in a wide variety of habitats. In the temperate zone of Europe, many darkling beetles and their larvae are bound to forested areas, saproxylic, feeding in decaying wood or/and on fungi and litter, but there is also a significant number of xerophilic species inhabiting dry environments. The English name ‘Darkling beetles’ is a translation of the scientific name of the family, referring to the generally uniformly brown to black colouration of European species. In the British fauna, there are 48 established species of darkling beetles known at present ( Duff, 2020).

The subfamily Alleculinae, known in English as comb-clawed beetles referring to the distinctly serrate pretarsal claws of adult beetles, most commonly develop in rotten wood, wood dust, or in soil, feeding on roots of various herbaceous plants or submerged decaying wood pieces. Adult comb-clawed beetles (except, for example, crepuscular and nocturnal *Hymenorus*, *Prionychus* etc.) are usually diurnal are commonly found visiting flowering herbaceous plants, shrubs and trees and feed on pollen and nectar. There are eight Alleculinae species in seven genera known in the British fauna ( Duff, 2020). The genus *Prionychus* Solier, 1835 is only present in the western part of the Palaearctic Region and comprises 12 species ( Novák, 2020), two of which are present also in the British fauna ( Duff, 2020). *Prionychus* differs from adults of other Palaearctic alleculine genera by the penultimate tarsomeres lobed ventrally.

Within the Alleculinae, *Prionychus ater* (Fabricius, 1775) is placed in the tribe Alleculini Laporte, 1840 ( Novák, 2020). The species is the type species of the genus *Prionychus*. Adults of the species are uniformly black to black-brown with paler tarsi, dorsally with short and inconspicuous greyish setae, appearing opaque or subopaque due to the microscopically reticulate pronotum and elytra and the comparatively shorter fourth antennomere, which is about twice as long as wide in this species ( Duff, 2020). Larvae are “mealworm”-like, narrow and strongly elongate, usually pale grey to yellowish with a comparatively darker head. Both imago and larvae dwell in hollow deciduous trees, under loose bark and in small cavities and cracks. Adults are crepuscular and nocturnal, usually found on tree trunks, occasionally flying to light. At present the two British species of *Prionychus* are difficult to separate, and may be confused in collections. It is hoped that genomic information may contribute towards a better understanding of generic concept of Alleculinae and to understanding of the origin of the British regional population of *Prionychus ater*.

*Prionychus ater* occurs in most of Europe, distributed from Portugal and the British Isles towards European Russia and Ukraine; the range extends to western Siberia in the East ( Novák, 2020). In the North it is present in southern Finland, southern Norway and southern portion of Sweden. It is recorded from 29 European countries, including the UK by Novák (2020), but is also present in Belgium, according to occurrence records on the GBIF (2025). The species is possibly also present in Bosnia and Herzegovina, Montenegro, North Macedonia and Serbia ( Telnov *et al.*, 2017). It is generally, much less abundant in the Iberian Peninsula, southern and southeastern Europe than in the zone of temperate forests and in the Baltic countries. Outside Europe, the species is known from the western part of Russian Siberia ( Novák, 2020). According to Telnov *et al*. (2017), the European extent of occurrence and area of occupancy of this species both strongly exceed the thresholds for a threatened species.

Originally a forest species, *Prionychus ater* is now also found in managed or artificial habitat – old parks, avenues and orchards. Larvae of *Prionychus ater* develop in brown or white tree rot and in boring dust produced by other insects which accumulates in hollows, cracks, cavities and under loose bark in live and dead trees ( Nikitsky *et al.*, 1996; Telnov *et al.*, 2017 and references therein). They prefer moderately wet substrate with large proportion of faeces produced by other saproxylic insects, but can also be observed in completely dry boring dust (Telnov, personal observations). Usually a small to very small (half of a cubic decimetre) amount of substrate is sufficient for a larva to develop. The species appears to have no specific preference for any tree species, and has been recorded from oaks ( *Quercus* spp.), willows ( *Salix* spp.), limes ( *Tilia* spp.), elm ( *Ulmus* spp.), horse chestnut ( *Aesculus* spp.), aspens ( *Populus* spp,), fruit trees ( Koch, 1989), rarely also Scots pine and spruce ( Telnov *et al.*, 2017). The larvae feed mostly on the remnants of dead insects and leaf litter, as well as on various decaying organics ( Nikitsky *et al.*, 1996). The species usually overwinters in the larval stage. Pupation occurs deeper in the substrate. The full development cycle in the northern and eastern parts of the range with continental or colder climate is no less than two years ( Nikitsky *et al.*, 1996; Telnov *et al.*, 2017). The bionomy of *Prionychus ater* in Central Europe is described as follows: adults are crepuscular, can be found from June to August in hollows of deciduous trees, larvae develop in decaying wood infested with polypore mycelia ( Novák, 2014).

*Prionychus ater* in the United Kingdom is locally distributed in southern and central England and very locally in SW England and Wales, and adults can be found from May to August ( Duff, 2020; GBIF Secretariat, 2025). The northernmost records known from north-eastern Yorkshire ( GBIF Secretariat, 2025). The species was not listed in the national Red Data Book ( Shirt, 1986), the present status of the species in the United Kingdom is Notable (B), meaning that it is estimated to occur in 31 to 100 of the 10 km squares of the UK National Grid ( Hyman, 1992). The species is uncommon and rarely seen as adult, but with some effort in the potential habitat, larvae can be found more easily than the adult beetles.

**Figure 1.**
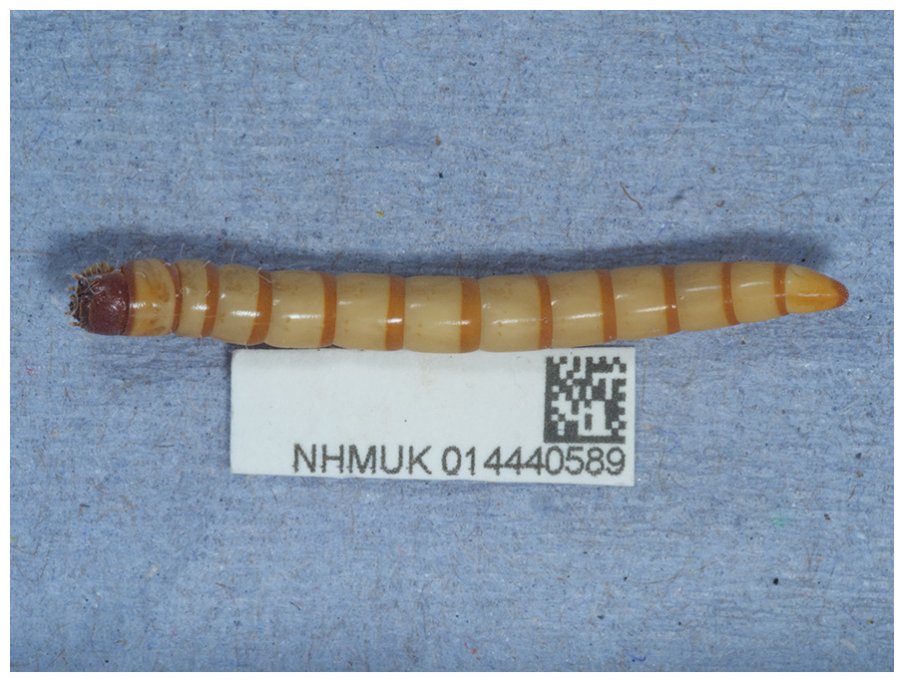
Photograph of the *Prionychus ater* (icPriAter1) specimen used for genome sequencing.

The assembly was produced using the Tree of Life pipeline from a specimen collected in Fulham Palace grounds, Fulham, London, England, United Kingdom ( Figure 1), as part of the Darwin Tree of Life project. It was identified by M.V.L. Barclay, and confirmed by comparing DNA barcode with adults from the UK deposited at the Natural History Museum, London. The specimen was collected from rotted segments of a large tree, also occupied by larvae of *Melanotus* sp. (Elateridae) and Lucanidae (either *Lucanus cervus* or *Dorcus parallelipipedus*). The grounds of Fulham Palace include ancient parkland, home to several exceptional trees, including a candidate for the earliest Holm Oak *Quercus ilex* in England, estimated at around 500 years old ( Hart, 2014). While there is no natural habitat nearby, the Palace grounds and the adjoining Bishops’ Park clearly provide sufficient dead wood to maintain populations of this and other saproxylic beetles.

## Methods

### Sample acquisition and DNA barcoding

The specimen used for sequencing was a large male larva of *Prionychus ater* (specimen ID NHMUK014440589, ToLID icPriAter1; Figure 1), collected from in the grounds of Fulham Palace, Fulham, London, England, United Kingdom (latitude 51.47, longitude –0.21) by S.V. Nikolaeva and M.V.L. Barclay on 2022-03-20. The same specimen was used for RNA sequencing.

The initial identification was verified by an additional DNA barcoding process according to the framework developed by Twyford *et al.* (2024). A small sample was dissected from the specimen and stored in ethanol, while the remaining parts were shipped on dry ice to the Wellcome Sanger Institute (WSI) (see the protocol). The tissue was lysed, the COI marker region was amplified by PCR, and amplicons were sequenced and compared to the BOLD database, confirming the species identification ( Crowley *et al.*, 2023). Following whole genome sequence generation, the relevant DNA barcode region was also used alongside the initial barcoding data for sample tracking at the WSI ( Twyford *et al.*, 2024). The standard operating procedures for Darwin Tree of Life barcoding are available on protocols.io.

Sample metadata were collected in line with the Darwin Tree of Life project standards described by Lawniczak *et al*. (2022).

### Nucleic acid extraction

Protocols for high molecular weight (HMW) DNA extraction developed at the Wellcome Sanger Institute (WSI) Tree of Life Core Laboratory are available on protocols.io ( Howard *et al.*, 2025). The icPriAter1 sample was weighed and triaged to determine the appropriate extraction protocol. Tissue from the abdomen was homogenised by powermashing using a PowerMasher II tissue disruptor. HMW DNA was extracted using the Automated MagAttract v2 protocol. We used centrifuge-mediated fragmentation to produce DNA fragments in the 8–10 kb range, following the Covaris g-TUBE protocol for ultra-low input (ULI). Sheared DNA was purified by automated SPRI (solid-phase reversible immobilisation). The concentration of the sheared and purified DNA was assessed using a Nanodrop spectrophotometer and Qubit Fluorometer using the Qubit dsDNA High Sensitivity Assay kit. Fragment size distribution was evaluated by running the sample on the FemtoPulse system. For this sample, the final post-shearing DNA had a Qubit concentration of 2.32 ng/μL and a yield of 301.60 ng. The 260/280 spectrophotometric ratio was 1.36, and the 260/230 ratio was 1.97.

RNA was also extracted from abdomen tissue of icPriAter1 in the Tree of Life Laboratory at the WSI using the RNA Extraction: Automated MagMax™ *mir*Vana protocol. The RNA concentration was assessed using a Nanodrop spectrophotometer and a Qubit Fluorometer using the Qubit RNA Broad-Range Assay kit. Analysis of the integrity of the RNA was done using the Agilent RNA 6000 Pico Kit and Eukaryotic Total RNA assay.

### PacBio HiFi library preparation and sequencing

Library preparation and sequencing were performed at the WSI Scientific Operations core. Prior to library preparation, the DNA was fragmented to ~10 kb. Ultra-low-input (ULI) libraries were prepared using the PacBio SMRTbell® Express Template Prep Kit 2.0 and gDNA Sample Amplification Kit. Samples were normalised to 20 ng DNA. Single-strand overhang removal, DNA damage repair, and end-repair/A-tailing were performed according to the manufacturer’s instructions, followed by adapter ligation. A 0.85× pre-PCR clean-up was carried out with Promega ProNex beads.

The DNA was evenly divided into two aliquots for dual PCR (reactions A and B), both following the manufacturer’s protocol. A 0.85× post-PCR clean-up was performed with ProNex beads. DNA concentration was measured using a Qubit Fluorometer v4.0 (Thermo Fisher Scientific) with the Qubit HS Assay Kit, and fragment size was assessed on an Agilent Femto Pulse Automated Pulsed Field CE Instrument (Agilent Technologies) using the gDNA 55 kb BAC analysis kit. PCR reactions A and B were then pooled, ensuring a total mass of ≥500 ng in 47.4 μl.

The pooled sample underwent another round of DNA damage repair, end-repair/A-tailing, and hairpin adapter ligation. A 1× clean-up was performed with ProNex beads, followed by DNA quantification using the Qubit and fragment size analysis using the Agilent Femto Pulse. Size selection was performed on the Sage Sciences PippinHT system, with target fragment size determined by Femto Pulse analysis (typically 4–9 kb). Size-selected libraries were cleaned with 1.0× ProNex beads and normalised to 2 nM before sequencing.

The sample was sequenced on a Revio instrument (Pacific Biosciences). The prepared library was normalised to 2 nM, and 15 μL was used for making complexes. Primers were annealed and polymerases bound to generate circularised complexes, following the manufacturer’s instructions. Complexes were purified using 1.2X SMRTbell beads, then diluted to the Revio loading concentration (200–300 pM) and spiked with a Revio sequencing internal control. The sample was sequenced on a Revio 25M SMRT cell. The SMRT Link software (Pacific Biosciences), a web-based workflow manager, was used to configure and monitor the run and to carry out primary and secondary data analysis.

### Hi-C

***Sample preparation and crosslinking***

The Hi-C sample was prepared from 20–50 mg of frozen head and thorax tissue of the icPriAter1 sample using the Arima-HiC v2 kit (Arima Genomics). Following the manufacturer’s instructions, tissue was fixed and DNA crosslinked using TC buffer to a final formaldehyde concentration of 2%. The tissue was homogenised using the Diagnocine Power Masher-II. Crosslinked DNA was digested with a restriction enzyme master mix, biotinylated, and ligated. Clean-up was performed with SPRISelect beads before library preparation. DNA concentration was measured with the Qubit Fluorometer (Thermo Fisher Scientific) and Qubit HS Assay Kit. The biotinylation percentage was estimated using the Arima-HiC v2 QC beads.

***Hi-C library preparation and sequencing***

Biotinylated DNA constructs were fragmented using a Covaris E220 sonicator and size selected to 400–600 bp using SPRISelect beads. DNA was enriched with Arima-HiC v2 kit Enrichment beads. End repair, A-tailing, and adapter ligation were carried out with the NEBNext Ultra II DNA Library Prep Kit (New England Biolabs), following a modified protocol where library preparation occurs while DNA remains bound to the Enrichment beads. Library amplification was performed using KAPA HiFi HotStart mix and a custom Unique Dual Index (UDI) barcode set (Integrated DNA Technologies). Depending on sample concentration and biotinylation percentage determined at the crosslinking stage, libraries were amplified with 10–16 PCR cycles. Post-PCR clean-up was performed with SPRISelect beads. Libraries were quantified using the AccuClear Ultra High Sensitivity dsDNA Standards Assay Kit (Biotium) and a FLUOstar Omega plate reader (BMG Labtech).

Prior to sequencing, libraries were normalised to 10 ng/μL. Normalised libraries were quantified again and equimolar and/or weighted 2.8 nM pools. Pool concentrations were checked using the Agilent 4200 TapeStation (Agilent) with High Sensitivity D500 reagents before sequencing. Sequencing was performed using paired-end 150 bp reads on the Illumina NovaSeq 6000.

### RNA library preparation and sequencing

Libraries were prepared using the NEBNext ^®^ Ultra™ II Directional RNA Library Prep Kit for Illumina (New England Biolabs), following the manufacturer’s instructions. Poly(A) mRNA in the total RNA solution was isolated using oligo(dT) beads, converted to cDNA, and uniquely indexed; 14 PCR cycles were performed. Libraries were size-selected to produce fragments between 100–300 bp. Libraries were quantified, normalised, pooled to a final concentration of 2.8 nM, and diluted to 150 pM for loading. Sequencing was carried out on the Illumina NovaSeq X to generate 150-bp paired-end reads.

### Genome assembly

Prior to assembly of the PacBio HiFi reads, a database of *k*-mer counts ( *k* = 31) was generated from the filtered reads using FastK. GenomeScope2 ( Ranallo-Benavidez *et al.*, 2020) was used to analyse the *k*-mer frequency distributions, providing estimates of genome size, heterozygosity, and repeat content.

The HiFi reads were assembled using Hifiasm in Hi-C phasing mode ( Cheng *et al.*, 2021; Cheng *et al.*, 2022), producing two haplotypes. Hi-C reads ( Rao *et al.*, 2014) were mapped to the primary contigs using bwa-mem2 ( Vasimuddin *et al.*, 2019). Contigs were further scaffolded with Hi-C data in YaHS ( Zhou *et al.*, 2023), using the --break option for handling potential misassemblies. The scaffolded assemblies were evaluated using Gfastats ( Formenti *et al.*, 2022), BUSCO ( Manni *et al.*, 2021) and MERQURY.FK ( Rhie *et al.*, 2020).

The mitochondrial genome was assembled using MitoHiFi ( Uliano-Silva *et al.*, 2023), which runs MitoFinder ( Allio *et al.*, 2020) and uses these annotations to select the final mitochondrial contig and to ensure the general quality of the sequence.

### Assembly curation

The assembly was decontaminated using the Assembly Screen for Cobionts and Contaminants ( ASCC) pipeline. TreeVal was used to generate the flat files and maps for use in curation. Manual curation was conducted primarily in PretextView and HiGlass ( Kerpedjiev *et al.*, 2018). Scaffolds were visually inspected and corrected as described by Howe *et al*. (2021). Manual corrections included 31 breaks and 319 joins. The curation process is documented at https://gitlab.com/wtsi-grit/rapid-curation. PretextSnapshot was used to generate a Hi-C contact map of the final assembly.

### Assembly quality assessment

The Merqury.FK tool ( Rhie *et al.*, 2020) was run in a Singularity container ( Kurtzer *et al.*, 2017) to evaluate *k*-mer completeness and assembly quality for both haplotypes using the *k*-mer databases ( *k* = 31) computed prior to genome assembly. The analysis outputs included assembly QV scores and completeness statistics.

The genome was analysed using the BlobToolKit pipeline, a Nextflow implementation of the earlier Snakemake version ( Challis *et al.*, 2020). The pipeline aligns PacBio reads using minimap2 ( Li, 2018) and SAMtools ( Danecek *et al.*, 2021) to generate coverage tracks. It runs BUSCO ( Manni *et al.*, 2021) using lineages identified from the NCBI Taxonomy ( Schoch *et al.*, 2020). For the three domain-level lineages, BUSCO genes are aligned to the UniProt Reference Proteomes database ( Bateman *et al.*, 2023) using DIAMOND blastp ( Buchfink *et al.*, 2021). The genome is divided into chunks based on the density of BUSCO genes from the closest taxonomic lineage, and each chunk is aligned to the UniProt Reference Proteomes database with DIAMOND blastx. Sequences without hits are chunked using seqtk and aligned to the NT database with blastn ( Altschul *et al.*, 1990). The BlobToolKit suite consolidates all outputs into a blobdir for visualisation. The BlobToolKit pipeline was developed using nf-core tooling ( Ewels *et al.*, 2020) and MultiQC ( Ewels *et al.*, 2016), with containerisation through Docker ( Merkel, 2014) and Singularity ( Kurtzer *et al.*, 2017).

## Genome sequence report

### Sequence data

**Figure 2.**
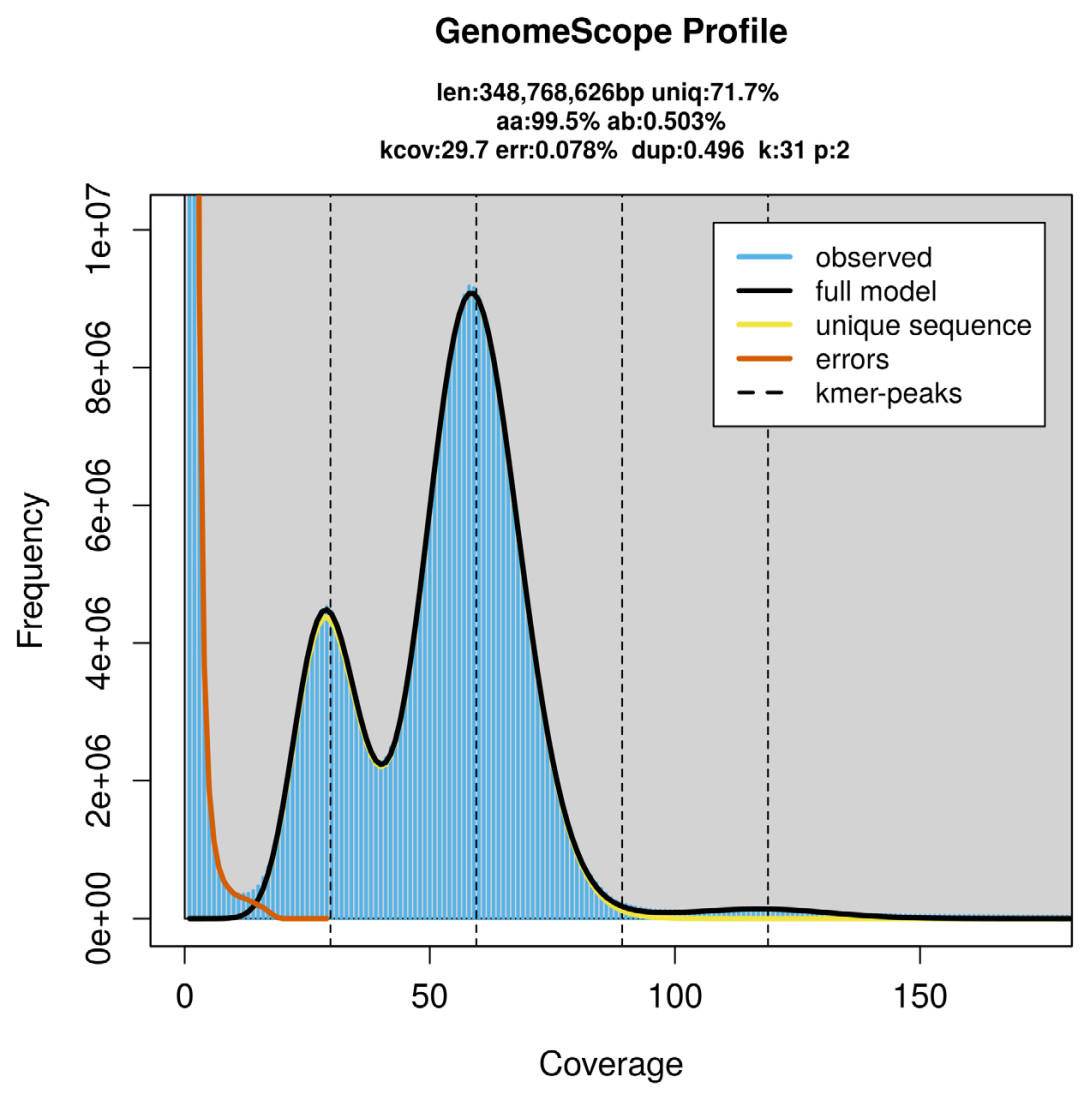
Frequency distribution of *k*-mers generated using GenomeScope2. The plot shows observed and modelled *k*-mer spectra, providing estimates of genome size, heterozygosity, and repeat content based on unassembled sequencing reads.

PacBio sequencing of the *Prionychus ater* specimen generated 21.95 Gb (gigabases) from 2.23 million reads, which were used to assemble the genome. GenomeScope2.0 analysis estimated the haploid genome size at 348.77 Mb, with a heterozygosity of 0.50% and repeat content of 28.33% ( Figure 2). These estimates guided expectations for the assembly. Based on the estimated genome size, the sequencing data provided approximately 59× coverage. Hi-C sequencing produced 124.89 Gb from 827.07 million reads, which were used to scaffold the assembly. RNA sequencing data were also generated and are available in public sequence repositories. Table 1 summarises the specimen and sequencing details.

**Table 1.** Specimen and sequencing data for BioProject PRJEB78953.

Platform	PacBio HiFi	Hi-C	RNA-seq
**ToLID**	icPriAter1	icPriAter1	icPriAter1
**Specimen ID**	NHMUK014440589	NHMUK014440589	NHMUK014440589
**BioSample (source individual)**	SAMEA112221776	SAMEA112221776	SAMEA112221776
**BioSample (tissue)**	SAMEA112221919	SAMEA112221850	SAMEA112221919
**Tissue**	abdomen	head and thorax	abdomen
**Instrument**	Revio	Illumina NovaSeq 6000	Illumina NovaSeq X
**Run accessions**	ERR13510342	ERR13621446	ERR14792844
**Read count total**	2.23 million	827.07 million	98.94 million
**Base count total**	21.95 Gb	124.89 Gb	14.94 Gb

### Assembly statistics

The genome was assembled into two haplotypes using Hi-C phasing. Haplotype 1 was curated to chromosome level, while haplotype 2 was assembled to scaffold level. The final assembly has a total length of 385.22 Mb in 360 scaffolds, with 413 gaps, and a scaffold N50 of 31.51 Mb ( Table 2).

**Table 2.** Genome assembly statistics.

Metric	Haplotype 1	Haplotype 2
**Assembly name**	icPriAter1.hap1.1	icPriAter1.hap2.1
**Assembly accession**	GCA_965112505.1	GCA_965112465.1
**Assembly level**	chromosome	scaffold
**Span (Mb)**	385.22	347.54
**Number of chromosomes**	14	N/A
**Number of contigs**	773	667
**Contig N50**	1.82 Mb	1.64 Mb
**Number of scaffolds**	360	234
**Scaffold N50**	31.51 Mb	31.7 Mb
**Longest scaffold length (Mb)**	37.51	N/A
**Sex chromosomes**	X	N/A
**Organelles**	Mitochondrion: 16.26 kb	N/A

**Figure 3.**
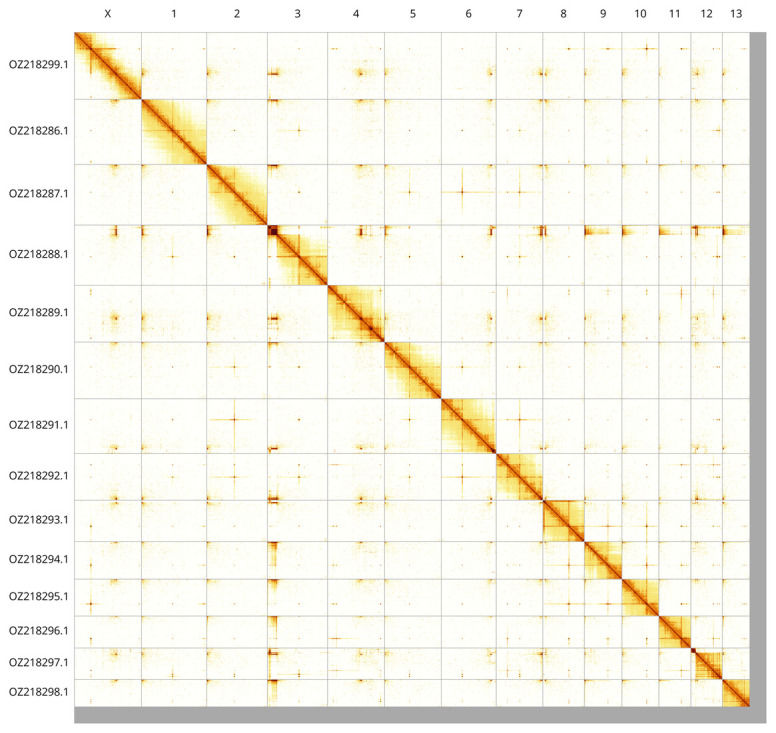
Hi-C contact map of the *Prionychus ater* genome assembly. Assembled chromosomes are shown in order of size and labelled along the axes. The plot was generated using PretextSnapshot.

Most of the assembly sequence (97.6%) was assigned to 14 chromosomal-level scaffolds, representing 13 autosomes and the X sex chromosome. These chromosome-level scaffolds, confirmed by Hi-C data, are named according to size ( Figure 3; Table 3). Chromosome X was identified by copy number in the diploid assembly. The species appears to be XO. The mitochondrial genome was also assembled. This sequence is included as a contig in the multifasta file of the genome submission and as a standalone record.

**Table 3.** Chromosomal pseudomolecules in the haplotype 1 genome assembly of *Prionychus ater* icPriAter1.

INSDC accession	Molecule	Length (Mb)	GC%
OZ218286.1	1	36.29	35.50
OZ218287.1	2	33.76	36
OZ218288.1	3	33.53	36.50
OZ218289.1	4	31.65	36.50
OZ218290.1	5	31.51	35.50
OZ218291.1	6	30.62	37
OZ218292.1	7	25.93	36
OZ218293.1	8	23.16	37
OZ218294.1	9	20.85	36.50
OZ218295.1	10	20.50	36.50
OZ218296.1	11	17.86	37
OZ218297.1	12	17.52	38
OZ218298.1	13	15.30	37.50
OZ218299.1	X	37.51	36

**Figure 4.**
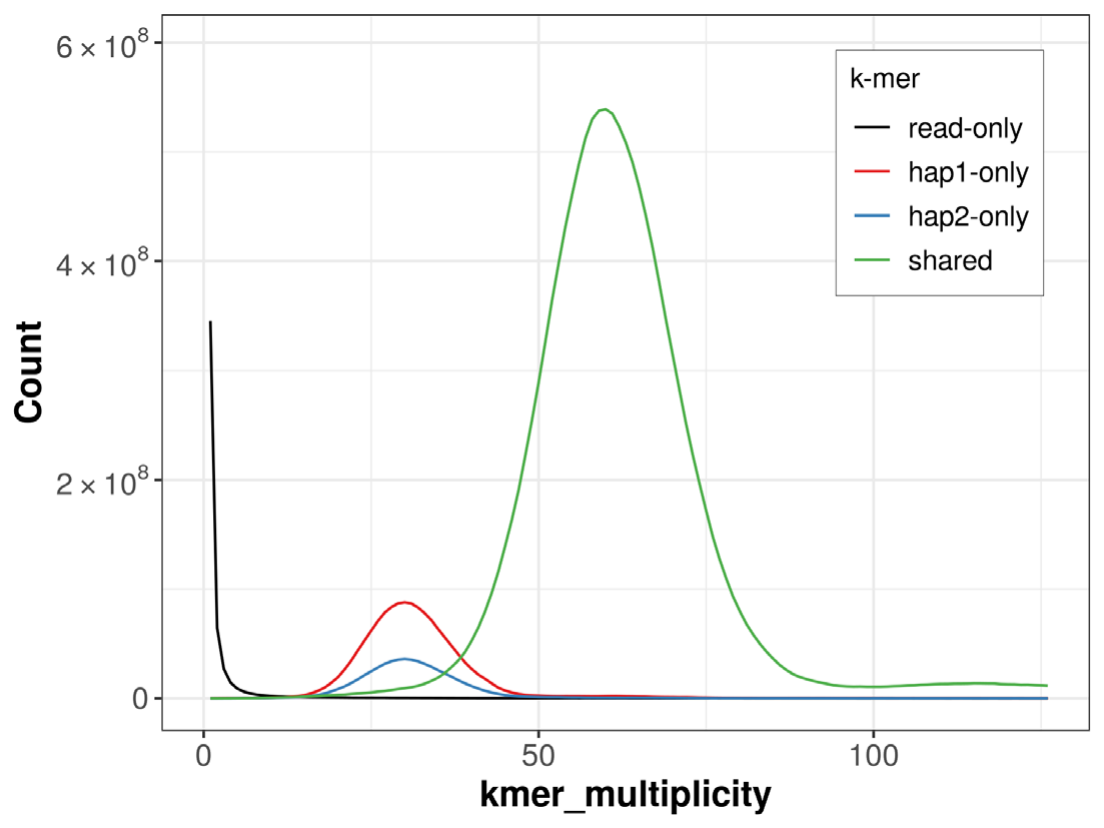
Evaluation of *k*-mer completeness using MerquryFK. This plot illustrates the recovery of *k*-mers from the original read data in the final assemblies. The horizontal axis represents *k*-mer multiplicity, and the vertical axis shows the number of *k*-mers. The black curve represents *k*-mers that appear in the reads but are not assembled. The green curve corresponds to *k*-mers shared by both haplotypes, and the red and blue curves show *k*-mers found only in one of the haplotypes.

For haplotype 1, the estimated QV is 60.7, and for haplotype 2, 61.1. When the two haplotypes are combined, the assembly achieves an estimated QV of 60.9. The *k*-mer completeness is 93.21% for haplotype 1, 83.98% for haplotype 2, and 99.63% for the combined haplotypes ( Figure 4).

**Figure 5.**
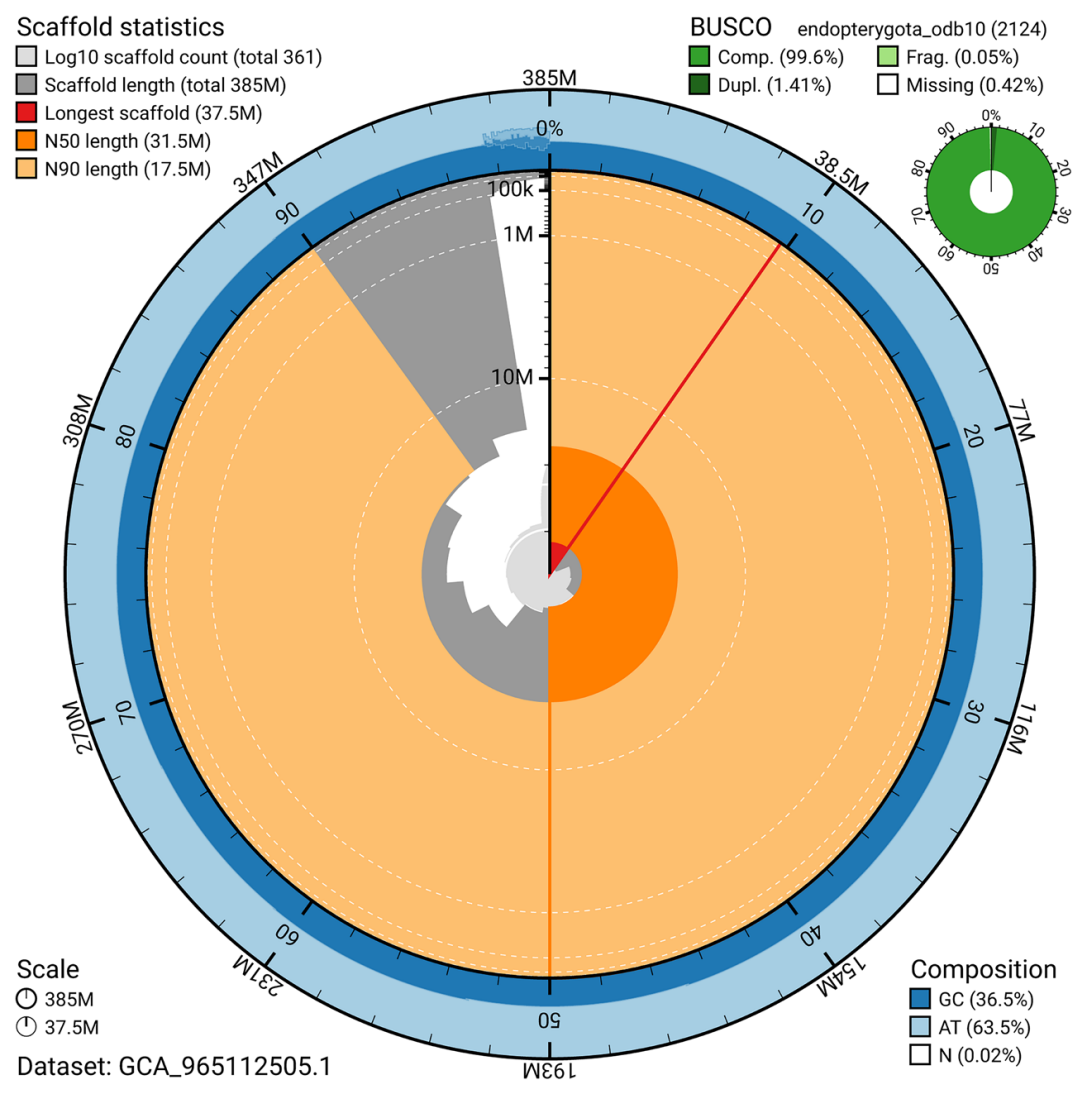
Assembly metrics for icPriAter1.hap1.1. The BlobToolKit snail plot provides an overview of assembly metrics and BUSCO gene completeness. The circumference represents the length of the whole genome sequence, and the main plot is divided into 1 000 bins around the circumference. The outermost blue tracks display the distribution of GC, AT, and N percentages across the bins. Scaffolds are arranged clockwise from longest to shortest and are depicted in dark grey. The longest scaffold is indicated by the red arc, and the deeper orange and pale orange arcs represent the N50 and N90 lengths. A light grey spiral at the centre shows the cumulative scaffold count on a logarithmic scale. A summary of complete, fragmented, duplicated, and missing BUSCO genes in the set is presented at the top right. An interactive version of this figure can be accessed on the BlobToolKit viewer.

**Figure 6.**
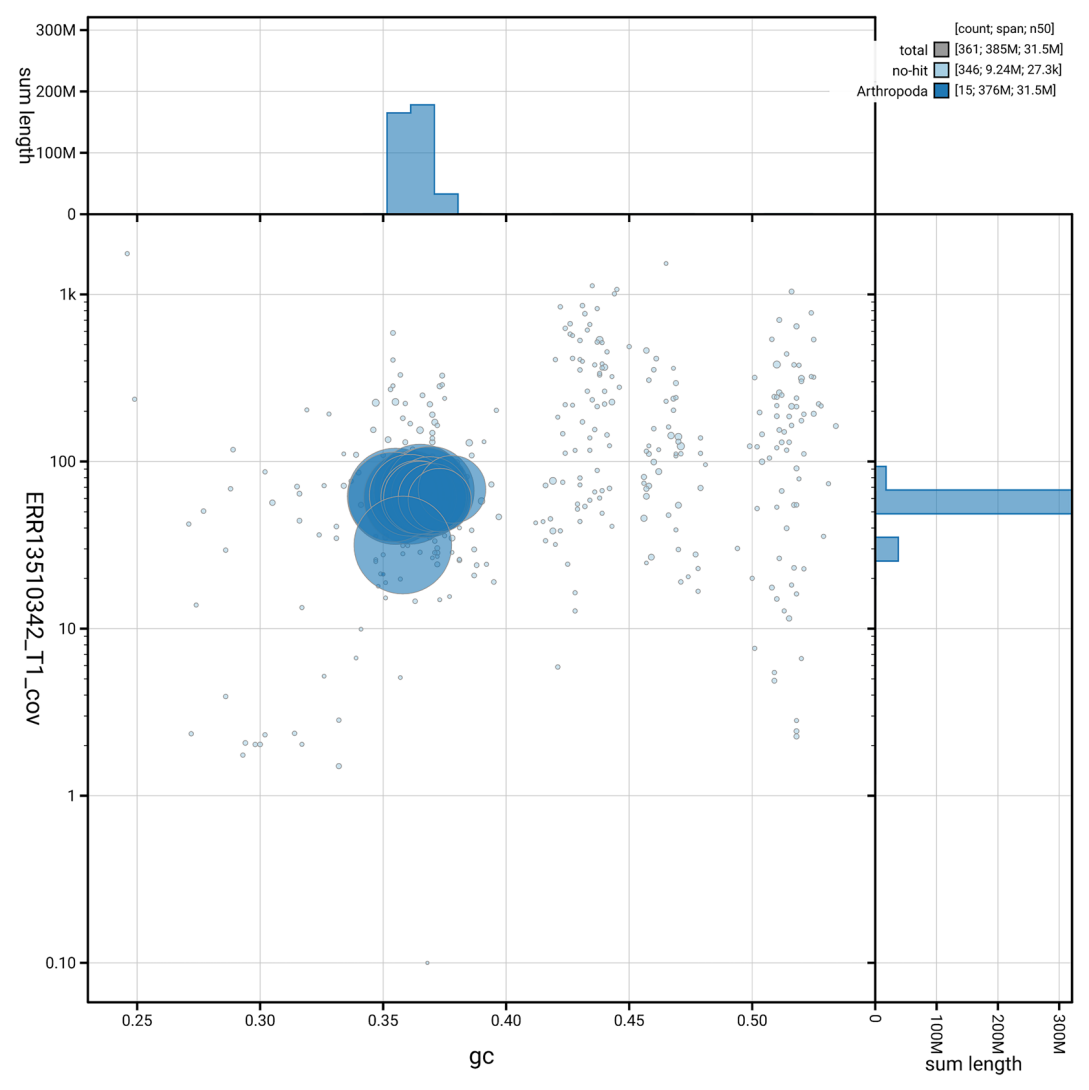
BlobToolKit GC-coverage plot for icPriAter1.hap1.1. Blob plot showing sequence coverage (vertical axis) and GC content (horizontal axis). The circles represent scaffolds, with the size proportional to scaffold length and the colour representing phylum membership. The histograms along the axes display the total length of sequences distributed across different levels of coverage and GC content. An interactive version of this figure is available on the BlobToolKit viewer.

BUSCO analysis using the endopterygota_odb10 reference set ( *n* = 2 124) identified 99.6% of the expected gene set (single = 98.2%, duplicated = 1.4%) for haplotype 1. The snail plot in Figure 5 summarises the scaffold length distribution and other assembly statistics for haplotype 1. The blob plot in Figure 6 shows the distribution of scaffolds by GC proportion and coverage for haplotype 1.

Table 4 lists the assembly metric benchmarks adapted from Rhie *et al.* (2021) the Earth BioGenome Project Report on Assembly Standards September 2024. The EBP metric, calculated for the haplotype 1, is **6.C.Q60**, meeting the recommended reference standard.

**Table 4.** Earth Biogenome Project summary metrics for the *Prionychus ater* assembly.

Measure	Value	Benchmark
EBP summary (haplotype 1)	6.C.Q60	6.C.Q40
Contig N50 length	1.82 Mb	≥ 1 Mb
Scaffold N50 length	31.51 Mb	= chromosome N50
Consensus quality (QV)	Haplotype 1: 60.7; haplotype 2: 61.1; combined: 60.9	≥ 40
*k*-mer completeness	Haplotype 1: 93.21%; Haplotype 2: 83.98%; combined: 99.63%	≥ 95%
BUSCO	C:99.6% [S:98.2%; D:1.4%]; F:0.0%; M:0.4%; n:2 124	S > 90%; D < 5%
Percentage of assembly assigned to chromosomes	97.60%	≥ 90%

### Wellcome Sanger Institute – Legal and Governance

The materials that have contributed to this genome note have been supplied by a Darwin Tree of Life Partner. The submission of materials by a Darwin Tree of Life Partner is subject to the **‘Darwin Tree of Life Project Sampling Code of Practice’**, which can be found in full on the Darwin Tree of Life website. By agreeing with and signing up to the Sampling Code of Practice, the Darwin Tree of Life Partner agrees they will meet the legal and ethical requirements and standards set out within this document in respect of all samples acquired for, and supplied to, the Darwin Tree of Life Project. Further, the Wellcome Sanger Institute employs a process whereby due diligence is carried out proportionate to the nature of the materials themselves, and the circumstances under which they have been/are to be collected and provided for use. The purpose of this is to address and mitigate any potential legal and/or ethical implications of receipt and use of the materials as part of the research project, and to ensure that in doing so we align with best practice wherever possible. The overarching areas of consideration are:

- Ethical review of provenance and sourcing of the material
- Legality of collection, transfer and use (national and international)

Each transfer of samples is further undertaken according to a Research Collaboration Agreement or Material Transfer Agreement entered into by the Darwin Tree of Life Partner, Genome Research Limited (operating as the Wellcome Sanger Institute), and in some circumstances, other Darwin Tree of Life collaborators.

## Data Availability

European Nucleotide Archive: Prionychus ater. Accession number PRJEB78953. The genome sequence is released openly for reuse. The *Prionychus ater* genome sequencing initiative is part of the Darwin Tree of Life Project (PRJEB40665) and the Sanger Institute Tree of Life Programme (PRJEB43745). All raw sequence data and the assembly have been deposited in INSDC databases. The genome will be annotated using available RNA-Seq data and presented through the Ensembl pipeline at the European Bioinformatics Institute. Raw data and assembly accession identifiers are reported in Table 1 and Table 2. Production code used in genome assembly at the WSI Tree of Life are available at https://github.com/sanger-tol. Table 5 lists software versions used in this study.

## Author information

Contributors are listed at the following links:

- Members of the Natural History Museum Genome Acquisition Lab
- Members of the Darwin Tree of Life Barcoding collective
- Members of the Wellcome Sanger Institute Tree of Life Management, Samples and Laboratory team
- Members of Wellcome Sanger Institute Scientific Operations – Sequencing Operations
- Members of the Wellcome Sanger Institute Tree of Life Core Informatics team
- Members of the Tree of Life Core Informatics collective
- Members of the Darwin Tree of Life Consortium
