# Supplementary material for: The genome sequence of the comb-clawed beetle, Prionychus ater (Fabricius, 1775) (Coleoptera: Tenebrionidae)
Source: Wellcome Open Res. 2025 Sep 8;10:483. [Version 1] doi: 10.12688/wellcomeopenres.24801.1 (PMC12529061; doi:10.12688/wellcomeopenres.24801.1)
Supplement: Peer review history

# Reviewer report: Reviewer response for version 1

**DOI:** 10.21956/wellcomeopenres.27324.r133902

**Published:** 2025-10-25

**Authors:** Hui Zhang

**Affiliations:** 1 Fujian Academy of Agricultural Sciences, Fujian, China

This manuscript presents a high-quality chromosome-level genome assembly of *Prionychus ater*, a significant contribution to the genomic resources for the diverse family Tenebrionidae. The study employs a comprehensive approach, utilizing PacBio HiFi long-read sequencing complemented by Hi-C and RNA-seq data, which has resulted in a haplotype-resolved assembly with excellent continuity (scaffold N50 of 31.51 Mb) and completeness (BUSCO score of 99.6%). The data presented are robust and will be a valuable resource for the scientific community.

 I have only a few minor comments and suggestions that could further enhance the clarity and completeness of the manuscript:

 The mitochondrial genome was assembled, but its accession number is not provided in the manuscript. I recommend including the accession number for the mitochondrial sequence, preferably in the main text or in Table 3.

 Please check and correct the grammar in the following sentence: “Adult tenebrionids are extremely heterogenous morphologically: nearly every unusual looking specimens one is not able to arrange to a family is highly likely to be a darkling beetle.”

Are sufficient details of methods and materials provided to allow replication by others?

Yes

Is the rationale for creating the dataset(s) clearly described?

Yes

Are the datasets clearly presented in a useable and accessible format?

Yes

Are the protocols appropriate and is the work technically sound?

Yes

Reviewer Expertise:

genomics, transcriptomics, evolutionary biology

I confirm that I have read this submission and believe that I have an appropriate level of expertise to confirm that it is of an acceptable scientific standard.

# Reviewer report: Reviewer response for version 1

**DOI:** 10.21956/wellcomeopenres.27324.r133898

**Published:** 2025-10-15

**Authors:** Aureliano Bombarely

**Affiliations:** 1 Institute for Molecular and Cellular Plant Biology (IBMCP, CSIC-UPV), Valencia, Spain

**Summary of the key results**

 The manuscript titled “The genome sequence of the comb-clawed beetle, Prionychus ater (Fabricius, 1775) (Coleoptera: Tenebrionidae)” describes the genome sequencing, and assembly of the darkling beetle species *Prionychus ater*. The assembly size was 385 Mb in 360 scaffolds. 97.6% of the assembly was anchored into the 14 expected pseudomolecules, including the X chromosome. The completeness was evaluated with BUSCO (99.6%) and Merqury (99.63% for both haplotypes).

**Overall evaluation**

 The assembly metrics looks fine. The methodology has been described in detail. There is not any concern about the presentation of the data.

**Discretionary changes**

- **More information about the quality of the DNA could be useful**. The authors specified the yield, and 260/280 and 260/230 ratio. It could be useful to know about the Nanodrop Concentration / Qbit Concentration ratio as well as fragment size.

Are sufficient details of methods and materials provided to allow replication by others?

Yes

Is the rationale for creating the dataset(s) clearly described?

Yes

Are the datasets clearly presented in a useable and accessible format?

Yes

Are the protocols appropriate and is the work technically sound?

Yes

Reviewer Expertise:

Genomics, Bioinformatics, Plant Evolution, Diversity, Agrogenomics.

I confirm that I have read this submission and believe that I have an appropriate level of expertise to confirm that it is of an acceptable scientific standard.

# Reviewer report: Reviewer response for version 1

**DOI:** 10.21956/wellcomeopenres.27324.r133901

**Published:** 2025-10-01

**Authors:** Anthony Bayega

**Affiliations:** 1 Canada's Michael Smith Genome Sciences Centre (Ringgold ID: 113898), Vancouver, British Columbia, Canada; 2 McGill University, Montreal, Canada

Crowley et al. provide a high quality chromosomal-level genome assembly of Anthracite Bee-fly, *Anthrax anthrax*, a commendable effort. I only have one comment on Figure 3; the label on the Y-axis which reads “ERR1…” could be changed to something more informative to help the reader make sense of this figure. Otherwise, the authors provide a good-quality genome and also assign a modest 87.88% of it to chromosomes. Although much work remains to order the scaffolds, fully phase the contigs and scaffolds, and complete the gaps and also structurally and functionally annotate the genome, the current work will indeed be valuable to the whole community.

Are sufficient details of methods and materials provided to allow replication by others?

Yes

Is the rationale for creating the dataset(s) clearly described?

Yes

Are the datasets clearly presented in a useable and accessible format?

Yes

Are the protocols appropriate and is the work technically sound?

Yes

Reviewer Expertise:

Genomics

I confirm that I have read this submission and believe that I have an appropriate level of expertise to confirm that it is of an acceptable scientific standard.
